# Pathomechanisms and biomarkers in facioscapulohumeral muscular dystrophy: roles of DUX4 and PAX7

**DOI:** 10.15252/emmm.202013695

**Published:** 2021-06-21

**Authors:** Christopher R S Banerji, Peter S Zammit

**Affiliations:** ^1^ Randall Centre for Cell and Molecular Biophysics King's College London London UK

**Keywords:** biomarker, DUX4, facioscapulohumeral muscular dystrophy (FSHD), pathology, PAX7, Genetics, Gene Therapy & Genetic Disease, Musculoskeletal System

## Abstract

Facioscapulohumeral muscular dystrophy (FSHD) is characterised by progressive skeletal muscle weakness and wasting. FSHD is linked to epigenetic derepression of the subtelomeric D4Z4 macrosatellite at chromosome 4q35. Epigenetic derepression permits the distal‐most D4Z4 unit to transcribe *DUX4*, with transcripts stabilised by splicing to a poly(A) signal on permissive 4qA haplotypes. The pioneer transcription factor DUX4 activates target genes that are proposed to drive FSHD pathology. While this toxic gain‐of‐function model is a satisfying “bottom‐up” genotype‐to‐phenotype link, DUX4 is rarely detectable in muscle and DUX4 target gene expression is inconsistent in patients. A reliable biomarker for FSHD is suppression of a target gene score of PAX7, a master regulator of myogenesis. However, it is unclear how this “top‐down” finding links to genomic changes that characterise FSHD and to DUX4. Here, we explore the roles and interactions of DUX4 and PAX7 in FSHD pathology and how the relationship between these two transcription factors deepens understanding via the immune system and muscle regeneration. Considering how FSHD pathomechanisms are represented by “DUX4opathy” models has implications for developing therapies and current clinical trials.

## Graphical abstract

In this review CRS Banerji and PS Zammit discuss the relationship between transcription factors DUX4 and PAX7 in the pathology of facioscapulohumeral muscular dystrophy.

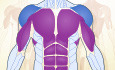

**Glossary**
B‐cell Acute Lymphoblastic Leukaemia (B‐ALL): A clonal malignant cancer characterised by blast cells phenotypically similar to stages of normal B‐cell differentiation. A subset of B‐cell acute lymphoblastic leukaemia cases express *DUX4* variants or a hybrid *DUX4‐IGH* fusion gene containing the DUX4 homeodomains.
D4Z4: A subtelomeric macrosatellite array of D4Z4 units at 4q35 and 10q26. Normally epigenetically repressed, truncation of the array and/or mutations in genes responsible for maintaining epigenetic repression, allows transcription of DUX4 from a retrotransposed open reading frame in the distal‐most D4Z4 unit.
DUX4 (DUX4‐fl): DUX4 (double homeobox 4) or DUX4‐fl (DUX4‐full length) is a 424 amino acid double homeodomain‐containing pioneer transcription factor encoded by each D4Z4 unit. DUX4 is involved in zygotic genome activation at the cleavage stage. Deregulated expression of DUX4 in somatic cells likely underlies FSHD.
DUX4‐s: A truncated 159 amino acid splice variant of DUX4 that is identical to DUX4 at the N‐terminus so contains the two homeodomains, but is shortened (hence DUX4‐s) and lacks the C‐terminal transactivation domain of DUX4.
DUX4c: A transcription factor largely similar to DUX4 but with a unique C‐terminus. Encoded by an inverted and truncated D4Z4 unit located approximately 42 kb centromeric of the D4Z4 array at 4q35.
DUX4 target gene signature: A biomarker of direct and indirect genes that are significantly activated by DUX4, usually derived by differential expression analysis in DUX4 over‐expression studies in human myogenic cells in vitro. Increased mean expression of all genes in a DUX4 target gene signature indicates that DUX4 is active, or was recently active, in the sample. Software is available to assess 3 DUX4 signatures at https://academic.oup.com/hmg/article/28/13/2224/5376488#supplementary‐data.
FSHD1: An inherited muscular dystrophy characterised by a descending skeletal muscle weakness and wasting that often displays left/right asymmetry. Caused by loss of D4Z4 units at 4q35 to between 1 and 10 on at least one allele leading to epigenetic derepression that permits transcription of *DUX4* from the distal‐most D4Z4 unit. *DUX4* transcripts are then stabilised and translated by splicing to a downstream poly(A) signal in the pLam region of permissive 4qA haplotypes.
FSHD2: Rare digenic variant of FSHD comprising ~5% of cases. Mainly caused by mutations in SMCHD1 that leads to epigenetic derepression at 4q35 when ~12–16 D4Z4 units are present on at least one allele. This permits transcription of DUX4 from the distal‐most D4Z4 unit that is then stabilised and translated due to splicing to a poly(A) signal on permissive 4qA haplotypes.
FSHD Lymphoblast score: A biomarker composed of 237 upregulated genes derived from immortalised FSHD B‐lymphoblastoid cell lines. Increased mean expression of all genes in the lymphoblast score distinguishes FSHD from control muscle biopsies and is strongest when MRI is used to guide biopsy selection.
PAX7: A paired‐homeobox transcription factor that is a master regulator of satellite cell specification and function in post‐natal and regenerative myogenesis.
PAX7 target gene signature: A biomarker of 311 up‐ and 290 downregulated direct and indirect PAX7 target genes derived by differential expression in primary murine satellite cell‐derived myoblasts over‐expressing Pax7 or a dominant‐negative Pax7 fusion protein. A single sample measure of PAX7 target gene activity is derived as the t‐statistic comparing mean expression of PAX7 upregulated to downregulated target genes. Software is available to assess the PAX7 signature at https://academic.oup.com/hmg/article/28/13/2224/5376488#supplementary‐data.
Satellite cell: The resident stem cell of skeletal muscle. Responsible for the routine needs of muscle homeostasis, together with the more sporadic demands for hypertrophy and repair. Normally mitotically quiescent in mature muscle, satellite cells can be activated and proliferate to generate myoblast progeny. Proliferative myoblasts then either self‐renew or differentiate, fusing to existing muscle fibres to provide new myonuclei or fusing together for *de novo* myotube/myofibre formation.
Zygotic Genome Activation (ZGA): Zygotes initially rely on maternal gene products to drive development, until chromatin re‐organisation permits zygotic genome activation. This switch from maternal to zygotic transcription proceeds in two phases of gene expression – a minor wave commencing in the 1 cell (1C) stage and a major wave from the mid‐to‐late 2C stage in mouse and 4‐8C in human.

## Introduction

Facioscapulohumeral muscular dystrophy (FSHD) is a prevalent autosomal dominant condition, characterised by progressive skeletal muscle weakness and wasting (Padberg, 1982; Wang & Tawil, 2016). Currently incurable, FSHD is associated with major morbidity and socioeconomic cost, with 20% of patients eventually becoming wheelchair dependent (Tawil & Van Der Maarel, 2006; Schepelmann *et al*, 2010).

There are two FSHD genetic subtypes: FSHD1 (OMIM: 158900) comprises ~95% of cases, with the remainder classified as FSHD2 (OMIM: 158901). Although genomic changes underlying FSHD1 and FSHD2 are distinct, the conditions are unified by ectopic expression of the full‐length isoform of the pioneer transcription factor *Double homeobox 4* (*DUX4*) (OMIM: 606009) (Dixit *et al*, 2007; Snider *et al*, 2010; Lemmers *et al*, 2012). Inappropriate expression of full‐length DUX4 (DUX4‐fl – henceforth called DUX4) and activation of its target genes is likely the critical molecular event driving FSHD pathology (Lemmers *et al*, 2010). Although this toxic gain‐of‐function model is a satisfying genotype‐to‐phenotype link, making DUX4 a clear therapeutic target, conclusively demonstrating the validity of the model is a stumbling block.

A consistent biomarker of FSHD is suppression of a target gene score of the transcription factor paired box 7 (PAX7) (OMIM: 167410) (Banerji *et al*, 2017; Banerji & Zammit, 2019), a master regulator of post‐natal myogenesis (Relaix & Zammit, 2012). Repression of this PAX7 target gene score reliably hallmarks FSHD, has a robust association with pathology and associates with disease progression (Banerji & Zammit, 2019; Banerji, 2020). Importantly, the homeodomains of DUX4 show homology with that of PAX7 and so DUX4 may interfere with the PAX7 transcriptional programme in FSHD (Bosnakovski *et al*, 2008; Banerji *et al*, 2017). PAX7 target gene signature repression has a strong link to the FSHD molecular phenotype, but connection to the FSHD genotype is underdeveloped.

Thus, the complexity of FSHD genetics and molecular pathology is not fully understood. Here, we discuss FSHD biomarkers and pathomechanisms, focussing on bottom‐up approaches centred on DUX4 and top‐down approaches aligned to PAX7, and consider how the relationship between these two transcription factors may explain some unanswered questions. We further reconcile these approaches via consideration of the immune system and muscle regeneration in FSHD. Understanding pathomechanisms underlying FSHD has clear implications for current clinical trials and developing novel therapies (Le Gall *et al*, 2020; Schatzl *et al*, 2021).

## Clinical features of FSHD

FSHD1 and FSHD2 form a disease continuum (Sacconi *et al*, 2019) characterised by a descending skeletal muscle weakness and wasting, often with marked left/right asymmetry (Padberg, 1982). FSHD patients also have an increased risk of chronic musculoskeletal pain (Jensen *et al*, 2008). FSHD typically presents in the second decade of life for males, earlier on average than in females (Zatz *et al*, 1998). There is also notable inter‐patient heterogeneity in symptom onset and severity, which can even occur in monozygotic twins (Tawil *et al*, 1993).

**Figure 1:**
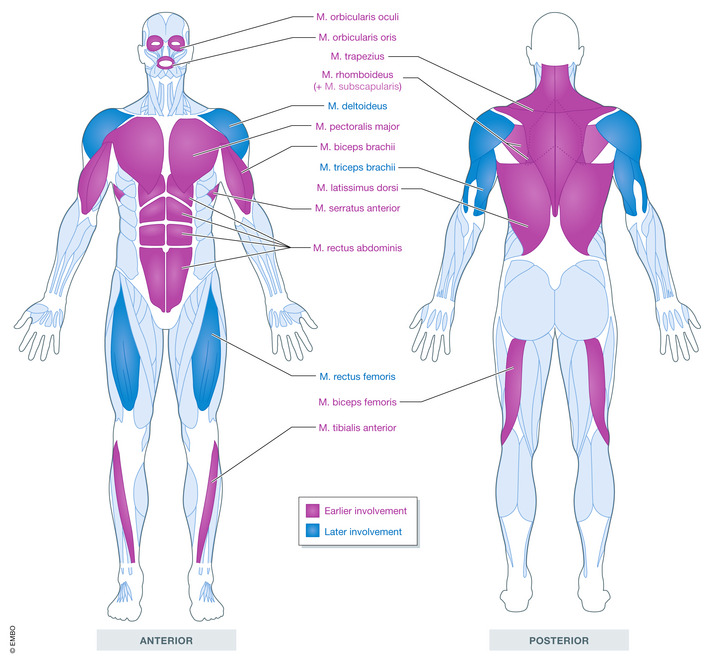
Early and late skeletal muscle involvement in FSHD Muscles/muscle groups typically affected in FSHD are colour‐coded so those that exhibit early involvement are illustrated in mauve, while those with later involvement are highlighted in blue.

The earliest clinical sign is often facial weakness in the orbicularis oculi and orbicularis oris (Fig 1), although such facial weakness is often reported later in life as it presents with typically unobtrusive symptoms, such as difficulty whistling and incomplete eye closure during sleep. The most common presenting symptom (~ 70% of patients) is weak shoulder abduction and scapular winging derived from weakness of latissimus dorsi, serratus anterior, pectoralis, subscapularis, rhomboids and, later, trapezius (Tyler & Stephens, 1950; Ricci *et al*, 2013; Tawil *et al*, 2014; Banerji *et al*, 2020a). The humeral component entails weakening of the biceps brachii, typically presenting at/after onset of shoulder girdle weakness. Curiously, deltoids and triceps brachii tend to be affected later in disease progression (Tyler & Stephens, 1950; Orrell, 2011) (Fig 1).

The term facioscapulohumeral does not fully encompass muscle involvement however. Following facial and upper limb weakness, classical presentations proceed to lower limbs, beginning around the mid/late 30s. Most characteristic is foot drop, resulting in a steppage gait due to weakness of the tibialis anterior and then hip girdle musculature (Tyler & Stephens, 1950). Again, neighbouring muscles are initially spared, such as quadriceps femoris. Lower limb effects can be severe, with patients needing ambulatory aids and wheelchairs (Hilbert *et al*, 2012; Eichinger *et al*, 2018). There is also abdominal muscle weakness, most notable in the inferior rectus abdominis (Eger *et al*, 2010) (Fig 1).

“Classical” FSHD describes approximately 68–75% of patients, with the remainder having atypical clinical presentations. Well documented is a facial sparing variant that associates with longer D4Z4 repeat length (Felice *et al*, 2000; Ricci *et al*, 2019). Rarer phenotypes include earlier lower limb muscle weakness, and milder involvement of facial and shoulder girdle muscles demonstrated in a UK population on the UK FSHD Patient Registry (Ricci *et al*, 2019; Banerji *et al*, 2020a) and confirmed in a US population on the National Registry for Myotonic Dystrophy (DM) and Facioscapulohumeral Dystrophy (FSHD) (unpublished data).

A number of extra‐muscular features are associated with FSHD, including a retinal vascular pathology resembling Coat’s disease, presenting as an arterial (rather than venous) tortuosity with micro‐aneurysmal changes (Goselink *et al*, 2019b). Although symptomatic retinal vascular disease is rare (0.2–1.5% of patients) (Tawil *et al*, 2015), a degree of aberrant retinal vasculature can be detected on fluorescence angiography in up to 75% of FSHD patients, associating inversely with D4Z4 repeat length (Fitzsimons, 2011; Goselink *et al*, 2019b). This may indicate wider vascular defects, and there is a reduced capillary density in FSHD muscle (Statland *et al*, 2015), as well as impaired muscle oxygenation (Olivier *et al*, 2016; Wilson *et al*, 2018). Sensorineural hearing loss is also a common extra‐muscular feature, with a prevalence of 12–19% (Tawil *et al*, 2015). Asymptomatic ECG abnormalities have been reported at varying prevalence, with the most notable being right bundle branch block (van Dijk *et al*, 2014).

Although overwhelmingly a late adolescence/adult onset disorder, ~ 5–10% of FSHD cases are infantile onset (< 10 years of age), typically with rapidly evolving disease along the classical trajectory (Goselink *et al*, 2019a). Some present with generalised hypotonia, and earlier onset of facial weakness associates with more severe disease (Mah *et al*, 2018). Increased prevalence of extra‐muscular features is also noted with infantile onset FSHD (Nikolic *et al*, 2016).

## Pathology in FSHD muscle

**Figure 2:**
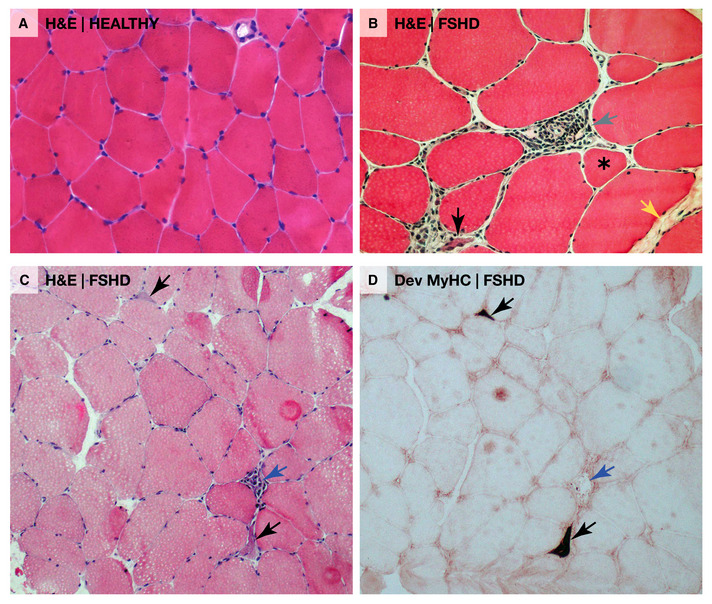
Microscopic muscle pathology in FSHD (A) Transverse section of skeletal muscle from a healthy adult stained with haematoxylin and eosin (H&E). (B) Transverse section of an FSHD skeletal muscle biopsy stained with haematoxylin and eosin showing hallmarks of FSHD, including rounded rather than polygonal shaped myofibres, increased endomysial fibrosis (yellow arrow), atrophic myofibres (asterisk), perivascular inflammation (grey arrow) and a small regenerating muscle fibre with a basophilic sarcoplasm due to increased RNA levels (black arrow). (C) FSHD muscle biopsy with a necrotic muscle fibre undergoing phagocytosis (blue arrow) and two basophilic regenerating muscle fibres (black arrows). (D) An adjacent section to that in (C) immunolabelled for developmental myosin heavy chain isoforms (Dev MyHC) using Novocastra NCL‐MHCd (Clone RNMy2/9D2). The two small myofibres with basophilic sarcoplasm identified in (C) contain developmental myosin heavy chains (black arrows), confirming that they are regenerating. Published with permission of Rabi N. Tawil.

**Figure 3:**
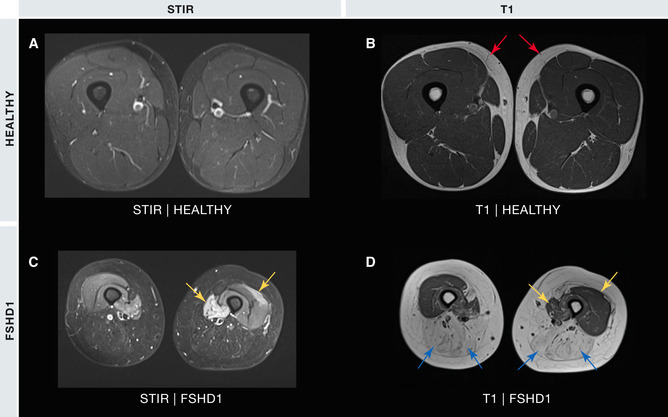
Macroscopic muscle pathology in FSHD MRI images from the thighs of a healthy 24‐year‐old male (A and B) and a 31‐year‐old female with FSHD1 (C and D), where white areas denote high signal intensity of the corresponding sequence. STIR images reveal a uniform signal across the thigh muscles of the healthy male (A). STIR images of the FSHD1 patient (C) display bright signals revealing muscle oedema/inflammation in parts of the vastus medialis and vastus lateralis (C – yellow arrows). The T1‐weighted image of the healthy muscle is uniformly low (B), contrasting with the high (white) signal from the subcutaneous fat (B – red arrows). Muscles of the posterior thigh of the FSHD1 patient (D) have a bright appearance using T1, indicative of fat replacement, including the semitendinosus, semimembranosus, gracilis adductor magnus and biceps femoris (D – blue arrows). The T1 signal remains dark in those portions of the vastus medialis and vastus lateralis of the FSHD1 patient that were bright with STIR imaging (compare C and D – yellow arrows), signifying that fat replacement has not occurred. Published with permission of Giorgio Tasca.

FSHD pathology shares features in common with most muscular dystrophies including muscle fibres that are atrophic and regenerating, that vary in size, exhibit central nucleation and/or are rounded, as well as endomysial fibrosis, fat infiltration and inflammation (Padberg, 1982; Wang & Tawil, 2016) (Fig 2). Endomysial and perivascular inflammation are prominent features of FSHD: detected microscopically via histopathology (Wang & Tawil, 2016) (Fig 2) and macroscopically via magnetic resonance imaging (MRI) using measures such as short tau inversion recovery (STIR) sequences (Fig 3) and decompositions of T2‐weighted signal intensity (Dahlqvist *et al*, 2020b; Felisaz *et al*, 2021). MRI measures of macroscopic inflammation are indicators of muscles at greater risk of fatty replacement as detected using T1‐weighted imaging (Fig 3), a feature correlated to clinical severity. However, these inflammatory markers are transient and fluctuating and, as a static measure, do not correlate to clinical severity (Monforte *et al*, 2019; Dahlqvist *et al*, 2020a). A small proportion of inflamed FSHD muscles also resolve without progression of fatty replacement, and fatty replacement can also occur in absence of inflammation, albeit at a slower rate. Inflammation in FSHD is thus sporadic and unpredictable, with potentially widespread non‐specific triggers in different muscle groups. Inflammation may therefore contribute to the left/right asymmetric muscle involvement that typifies FSHD.

## Genetic basis of FSHD

FSHD1 is associated with a partial deletion in the D4Z4 macrosatellite repeat array in the subtelomere of at least one chromosome 4q35 allele (Himeda & Jones, 2019; Greco *et al*, 2020). There are usually 11 to >100 D4Z4 tandem repeats arranged in a head‐to‐tail configuration in unaffected individuals, but FSHD1 is characterised by having only 1–10 units (Wijmenga *et al*, 1992; van Deutekom *et al*, 1993). Macrosatellite length generally inversely correlates to disease onset and severity. D4Z4 unit reduction leads to epigenetic derepression at the locus, including DNA hypomethylation and chromatin relaxation, although the extent is not strictly dictated by repeat length (van Overveld *et al*, 2003; Lemmers *et al*, 2015; Himeda & Jones, 2019; Salort‐Campana *et al*, 2020).

FSHD2 is digenic. Loss of D4Z4 repeats at 4q35, with 12–16 D4Z4 units typical, is coupled with mutations in proteins that maintain epigenetic repression at D4Z4. In > 90% of FSHD2 patients, this mutation is in *structural maintenance of chromosomes flexible hinge domain containing 1 (SMCHD1* – OMIM: 614982) (Lemmers *et al*, 2012). Much more rarely, FSHD2 is associated with mutations in *DNA methyltransferase 3 beta* (*DNMT3B)* (van den Boogaard *et al*, 2016) and, in a single case, *ligand‐dependent nuclear receptor‐interacting factor 1 (LRIF1)* (Hamanaka *et al*, 2020).

SMCHD1 is involved in methylation, silencing and compaction of the inactive X chromosome and repression at specific autosomal loci (Gendrel *et al*, 2013; Mason *et al*, 2017). Interestingly, heterozygous *SMCHD1* mutations cause the congenital syndrome Bosma arhinia microphthalmia, characterised by severe hypoplasia of nose and eyes, palatal abnormalities, inguinal hernias and hypogonadotropic hypogonadism with cryptorchidism in males (Shaw *et al*, 2017). The non‐overlap in clinical phenotypes between FSHD2 and Bosma arhinia microphthalmia is likely due to different disease‐specific residues (Lemmers *et al*, 2019). *SMCHD1* mutations also act as a disease modifier in FSHD1, implying an additive effect of FSHD1 and FSHD2 genotypes (Sacconi *et al*, 2013; Sacconi *et al*, 2019).

Crucially, at least one D4Z4 unit is required for FSHD (Tupler *et al*, 1996). Each 3.3 kb D4Z4 unit contains a retrotransposed promoter and a single open reading frame encoding *DUX4* (Hewitt *et al*, 1994; Gabriels *et al*, 1999). *DUX4* is repressed in somatic cells via epigenetic modification at D4Z4 (Himeda & Jones, 2019). In FSHD, the genomic changes drive epigenetic derepression at D4Z4, permitting *DUX4* transcription from the distal‐most D4Z4 unit (Dixit *et al*, 2007). *DUX4* gene transcription initiates at its promoter in the distal D4Z4 unit to include coding exon 1 and non‐coding exon 2, and then extends into the 3' flanking region comprising one of two equally common 4qA or 4qB allelic forms. The 4qA haplotype is characterised by the pLAM sequence, containing a poly(A) signal on permissive haplotypes, and a telomeric 68 bp β‐satellite array. For translation, *DUX4* transcripts require *cis* splicing to this poly(A) signal in the non‐coding exon 3 (Lemmers *et al*, 2010). 4qA haplotypes are classified on simple sequence length polymorphisms (SSLPs) proximal to the D4Z4 repeats (Lemmers *et al*, 2007), with the 4A161 SSLP variant and rare 4A159 and 4A168 all associated with FSHD. Thus, both FSHD1 and FSHD2 require epigenetic derepression of D4Z4 and a permissive 4qA haplotype containing a poly(A) signal (Himeda & Jones, 2019; Greco *et al*, 2020).

## Gene modifiers in FSHD

Notably, a permissive genomic configuration does not guarantee FSHD pathology, with a significant proportion of carriers in FSHD families not manifesting disease. Moreover, 1.3% of healthy individuals unrelated to any FSHD patients have a “pathogenic” allele (defined as 4–8 D4Z4 repeats and permissive 4qA161 haplotype) (Scionti *et al*, 2012). Of relevance, although the single D4Z4‐2.5 mouse line transgenic for a 2.5 D4Z4 repeat unit from an FSHD patient displayed epigenetic features at the human D4Z4 locus, there was no muscular dystrophic phenotype (Krom *et al*, 2013). Thus, further modifiers are needed for disease penetrance.

Approximately 42 kb centromeric of the D4Z4 array is an inverted and truncated D4Z4 unit that encodes DUX4c, a double homeodomain transcription factor largely identical to DUX4, but with a unique C‐terminus (Ansseau *et al*, 2009). Epigenetic derepression at D4Z4, therefore, might also affect regulation of *DUX4c* (Ansseau *et al*, 2009). Similarly, regulation of other genes located centromeric to D4Z4 may be also be affected, with mouse models over‐expressing *Frg1* (Gabellini *et al*, 2006)*, Slc25a4* (*Ant1*) (preprint: Wang *et al*, 2020) or null mutations in *Fat1* (Caruso *et al*, 2013) developing muscular dystrophic phenotypes with some similarities to FSHD. Such genes may act as genetic modifiers in FSHD, affecting onset, muscle selection and/or severity (Park *et al*, 2018).

D4Z4 location near telomeres could also facilitate mis‐regulation of DUX4, as telomeric shortening associates with epigenetic changes and increased *DUX4* expression (Stadler *et al*, 2013), providing a mechanism for non‐genetic heterogeneity, asymmetric muscle weakness and adult onset in FSHD. Telomere shortening and the modified chromatin landscape at 4q35 in FSHD could also affect expression of other genes in the region including *DUX4c*,*FRG1, FRG2* and *FAT1* in acting as gene modifiers (Gaillard *et al*, 2019). Such gene modifiers could also be influenced by long non‐coding (lnc) RNA. Epigenetic changes in the region centromeric to the D4Z4 array permit transcription of *DBE‐T*, a nuclear lncRNA that associates with local chromatin. *DBE‐T* binds and recruits the trithorax protein ASH1L to add histone methylation marks that facilitate gene activation in the region (Cabianca *et al*, 2012).

Very rare cases (2 families) of FSHD associated with a reduced number of D4Z4 repeats located in the subtelomere of 10q from *de novo* D4Z4 repeat exchange between chromosomes 4 and 10, indicate that D4Z4 units encoding *DUX4* and a poly(A) signal are essential for pathology. However, the *FRG2* gene remains present at 10q in these patients and is upregulated, and so involvement of FRG2 in pathology cannot be excluded (Lemmers *et al*, 2010; Lemmers *et al*, 2021). The potential roles of *FRG1* and *FRG2* are further complicated as both are direct targets genes of DUX4 (Thijssen *et al*, 2014; Ferri *et al*, 2015).

## DUX4 evolution and transcription

Genomic alterations in FSHD clearly implicate DUX4 (Himeda & Jones, 2019), leading to a statement in most recent FSHD publications to the effect: “mis‐expression of DUX4 underlies FSHD pathology”, and generally highlighting DUX4 as a therapeutic target. However, better understanding is needed as to the role of DUX4 in health and its contribution to non‐FSHD disorders, in addition to whether *DUX4* expression directly contributes to ongoing pathology and so is practical to target as an FSHD therapy.

Double homeobox (DUX) transcription factors are unique to placental mammals (Leidenroth & Hewitt, 2010). Each DUX homeodomain contains an ancient coding sequence found in single homeodomain proteins in fungi, plants and animals (Leidenroth & Hewitt, 2010). A duplication event likely generated the first *DUX* gene, the intron‐containing *DUXC*, present in the most recent common ancestor of placental mammals (Leidenroth *et al*, 2012). Subsequent retrotransposition and displacement of *DUXC* produced the *DUX* family in human, including *DUX4* arrays in the subtelomeres at 4q and 10q (Gabriels *et al*, 1999; Leidenroth *et al*, 2012). Importantly, conservation of a *DUX4* coding region in apes and Old World monkeys (Clark *et al*, 1996) and *DUX4*‐like genes in other mammalian species (Leidenroth *et al*, 2012) indicate a conserved function.

The two DUX4 homeodomains bind different target DNA sequences (homeodomain 1: 5‐TAAT‐3; homeodomain 2: 5‐TGAT‐3) in a head‐to‐head configuration (Lee *et al*, 2018). DUX4 not only binds DNA primed for transcription by being DNase accessible with H3K27Ac‐rich chromatin, but as a pioneer transcription factor, can also bind DNase inaccessible H3K27Ac‐depleted MaLR‐enriched chromatin. This is achieved by DUX4‐mediated recruitment of the histone acetyltransferases p300/CREB‐binding protein (CBP) to reorganise H3K27 acetylation at target genes to promote their transcriptional activation (Choi *et al*, 2016b).

Transcription of *DUX4* is complex and five splice variants encode two proteins: the full‐length 424 amino acid DUX4 protein (aka DUX4‐fl) and a truncated species containing the N‐terminal 159 amino acids including both homeodomains called DUX4‐s, that lacks the C‐terminal transactivation domain (Snider *et al*, 2009; Sidlauskaite *et al*, 2020). Generating mutant DUX4 proteins reveals that the homeodomains and C‐terminal most 25 amino acids are sufficient for most DUX4 activity (Bosnakovski *et al*, 2017b; Mitsuhashi *et al*, 2018). Post‐transcriptional repression of mis‐expressed *DUX4*‐*fl* may be achieved via splicing into *DUX4‐s*, which is found in healthy individuals and is non‐pathogenic (Snider *et al*, 2010), and unable to drive the majority of transcriptional changes induced by DUX4 in mouse (Knopp *et al*, 2016).

## DUX4 function in health

DUX4 plays a recently discovered role in zygotic genome activation (ZGA). Zygotes initially rely on maternal gene products to drive development, until chromatin re‐organisation permits ZGA. This switch from maternal to zygotic transcription proceeds in two phases of gene expression – a minor wave commencing at the 1 cell (1C) stage and a major wave from the mid‐to‐late 2C stage in mouse and 4‐8C in human. *DUX4* transcripts are detected between 2C and 4C during the major wave of ZGA, where p300/CBP recruitment likely facilitates chromatin opening, before peaking at 8C (De Iaco *et al*, 2017; Hendrickson *et al*, 2017; Whiddon *et al*, 2017).

This role of DUX4 in ZGA requires *DUX4* transcripts to be stabilised in healthy individuals lacking permissive 4qA or 10q haplotypes. There is no clear mechanism in somatic cells, but stabilisation may occur via an exon 7 poly(A) signal accessible in germ cells (Snider *et al*, 2010).

*mDUX* is the murine ortholog of *DUX4* and displays a similar expression pattern to *DUX4* (Hendrickson *et al*, 2017), but while e*x vivo* CRISPR/Cas9‐mediated knockout of *mDUX* arrests embryos at the 2C stage and prevents ZGA (De Iaco *et al*, 2017), some homozygous *mDUX^−/−^* mice can develop relatively normally, with only minor delays in ZGA (Chen & Zhang, 2019; De Iaco *et al*, 2020; Bosnakovski *et al*, 2021). Transient over‐expression of *mDUX* increases efficiency of somatic nuclear transfer, as well as chemical induction of induced pluripotent stem cells, via induction of ZGA (Yang *et al*, 2020).

*DUX4* remains expressed in the germ line, particularly testis, irrespective of 4qA status by using the poly(A) signal in exon 7 (Snider *et al*, 2010; Young *et al*, 2013). DUX4 is silenced in somatic cells by location within macrosatellite regions at the 4q and 10q subtelomeres (Himeda & Jones, 2019). Despite evidence for somatic repression, DUX4 has been proposed to play a role in osteogenic and keratinocyte differentiation. DUX4 and a 5’ extended long species are detectable during osteogenic differentiation and knockdown inhibits osteogenesis (de la Kethulle de Ryhove *et al*, 2015). In late differentiating keratinocytes, H3K9me3 changes at the D4Z4 region at 4q and 10q associate with expression of two DUX4 protein variants including DUX4‐fl, and activation of DUX4 target genes (e.g. ZSCAN4, TRIM43, DEFB103). This is proposed to regulate apoptosis and keratinocyte late‐terminal differentiation (Gannon *et al*, 2016). Interestingly, some DUX4 over‐expressing mouse models develop hyper‐keratotic skin phenotypes and alopecia (Krom *et al*, 2013; Dandapat *et al*, 2014). *DUX4* expression has also been found in the thymus of healthy individuals (Das & Chadwick, 2016). This may be a consequence of antigen presentation to promote central tolerance during T‐cell maturation. However, given that DUX4 induces expression of immune system‐related genes, thymus expression may be indicative of a role for DUX4 in lymphocyte development (Das & Chadwick, 2016).

## DUX4 in non‐FSHD pathologies

Involvement of DUX4 in non‐FSHD pathologies gives insight into how DUX4 drives pathology in FSHD, as well as informing about DUX4 function and effects in non‐muscle tissues. This may also extend the scope for anti‐DUX4 therapies.

DUX4 upregulates target genes in several epithelial malignancies. Intriguingly, while there is correlation between *DUX4* expression and permissive 4qA haplotypes in tumours, it is not absolute (Chew *et al*, 2019). DUX4 may promote immune evasion in cancer by blocking interferon‐γ‐regulated MHC class I genes, so reducing antigen presentation to CD8^+^ T cells. Indeed, *DUX4* expression is associated with resistance to anti‐CTLA‐4 immunotherapy in melanoma (Chew *et al*, 2019). Interestingly, the bulk tumour RNA‐seq deconvolution algorithm TIMER (Li *et al*, 2017) revealed a lower proportion of CD8^+^ T and natural killer cells in DUX4‐expressing tumours, in line with evasion of tumour immune surveillance.

DUX4 protein is also found in cells of the synovial lining layer and underlying connective tissue in synovial tissue from patients with rheumatoid arthritis and the autoimmune axial spondyloarthritis, characterized by chronic inflammation (Quaden *et al*, 2020).

Several pathologies involve translocation of part of the *DUX4* coding region to another promoter. A subset of B‐cell acute lymphoblastic leukaemia (B‐ALL) cases is associated with D4Z4 insertions in the *IGH* (and rarely *ERG*) locus in both orientations and with various reading frames, leading to expression of truncated DUX4 variants or hybrid DUX4‐IGH fusion proteins containing the DUX4 homeodomains (Lilljebjorn *et al*, 2016; Yasuda *et al*, 2016). DUX4‐IGH can arrest B‐cell differentiation and induce transformation (Yasuda *et al*, 2016; Dong *et al*, 2018). Curiously, while DUX4‐IGH binds DUX4 response elements (Dong *et al*, 2018), it does not induce as strong a transcriptional response as DUX4 (149 genes versus 1,519 with DUX4). The C‐terminal transactivation domain of *DUX4* is fused to the *CIC* gene in ~ 27% of Ewing sarcoma, but without the homeodomains, the CIC‐DUX4 fusion protein cannot activate DUX4 target genes (Kawamura‐Saito *et al*, 2006).

Although similarities in differentially expressed genes have been described in FSHD and cancer biopsies, a consensus direction of gene expression is lacking, and it is unclear whether cancer and FSHD are positively or negatively transcriptomically associated (Dmitriev *et al*, 2014). However, genealogical analysis does not show any clinical or epidemiological association between FSHD and cancer in FSHD1 patients from 31 families (Dr Peter Lunt: personal communication).

## Working from the bottom up: genotype to phenotype

A bottom‐up approach establishes a genotype‐to‐phenotype link. If DUX4 is key, how does it cause pathological damage in FSHD muscle? The first point to establish is the expression dynamics of *DUX4* in skeletal muscle.

Most FSHD patients have two enhancers (DME1 and DME2) proximal to the D4Z4 region (Himeda *et al*, 2014) that likely augment *DUX4* expression in zygotes. Epigenetic changes at the locus in FSHD may reveal binding sites in DME1/2 for myogenic regulatory factors and PAX proteins such as PAX3 and PAX7, which could then facilitate *DUX4* expression in myofibres and muscle stem cells (satellite cells). Motifs for other DNA‐binding factors also give the potential for driving *DUX4* in a range of somatic cell types (Himeda *et al*, 2014).

However, detection of *DUX4* transcripts and protein in FSHD patient muscle biopsies is notoriously difficult (Dixit *et al*, 2007; Snider *et al*, 2010; Vanderplanck *et al*, 2011). While highly sensitive nested RT–qPCR has detected *DUX4* in foetal and adult FSHD muscle biopsies in some studies (Jones *et al*, 2012; Broucqsault *et al*, 2013), multiple RNA‐seq studies have not (Yao *et al*, 2014; Wang *et al*, 2019; Wong *et al*, 2020). On the individual cell/nuclear level, *DUX4* was detected by scRNA‐seq in *~ *0.5*%* of myocytes (myoblasts that differentiate without fusing into immature, multinucleated muscle fibres called myotubes) *ex vivo*, pooled from four patients (van den Heuvel *et al*, 2019) (Table 1). SnRNA‐seq identified *DUX4* transcripts in 3.8% of muscle fibre myonuclei *ex vivo* from one FSHD2 patient, but no transcripts were detected in a second patient (Table 1). RNA fluorescence in situ hybridisation of the *DUX4*‐positive FSHD2 patient showed ~ 7% of myotubes expressed *DUX4*, with a mean of 2/15 *DUX4*‐positive nuclei per myotube (Jiang *et al*, 2020).

Technical issues may contribute to poor detection. *DUX4‐fl* transcripts are GC‐rich (70.6% in the major transcript) and such transcripts are generally read sparse in RNA‐seq using Illumina technologies, with A‐T residues sequenced with a higher frequency than G‐C (Price *et al*, 2017). Furthermore, older alignment methodologies do not directly account for GC bias, while newer methodologies do, such as Salmon (Patro *et al*, 2017). GC‐rich regions in primer and template can also cause competitive annealing and inaccurate results with RT–qPCR (Mamedov *et al*, 2008).

DUX4 protein has been reported in FSHD muscle biopsies via Western blot, but not in more affected muscles, implying expression may be an early, transient event in pathogenesis (Tassin *et al*, 2013). DUX4 protein has not been reliably shown by immunolabelling FSHD muscle, so cellular/tissue distribution is unknown. FSHD myoblasts *ex vivo* have very low levels of DUX4 protein (~0.001% cells), but this increases during differentiation (0.05% myotube nuclei) (Snider *et al*, 2010; Rickard *et al*, 2015) consistent with expression of transcription factors able to bind the *DUX4* DME1/2 enhancers (Himeda *et al*, 2014).

## How can such low level DUX4 expression drive pathology?

*DUX4* expression is stochastic, with bursts in very few nuclei at any given time (Rickard *et al*, 2015). A DUX4 reporter showed DUX4 activity peaking in 0.29–4.28% of cells after 48–54 h of differentiation, with positive cells then dying on average 20 h later (Rickard *et al*, 2015). Gradients of DUX4 in syncytial myotubes imply that DUX4 emanating from a single nucleus could induce transcriptional change in many neighbouring myonuclei (Tassin *et al*, 2013; Ferreboeuf *et al*, 2014; Jiang *et al*, 2020). There is also the intriguing possibility that DUX4 may be able to cross cell membranes, by virtue of the homeodomains, to have effects in adjacent cells (Lee *et al*, 2019).

Amongst DUX4 target genes are transcription factors including DUXA and LEUTX that could potentiate DUX4 target gene activation (Jiang *et al*, 2020; Chau *et al*, 2021). DUX4 also operates via modifying chromatin by inducing expression of the histone variants H3.X and H3.Y, that then incorporate into DUX4 target genes to promote to greater perdurance and improved reactivation (Resnick *et al*, 2019).

Control of DUX4 is auto‐regulatory via DUX4‐triggered degradation of UPF1, a central component of the nonsense‐mediated decay machinery (Feng *et al*, 2015). Conversely, DUX4 is degraded by the ubiquitin–proteasome pathway (Tassin *et al*, 2013). Thus, bursts of DUX4 are quickly dampened, resulting in low detection, but this may belie higher cumulative *DUX4* expression.

The binding motifs for PAX3 and PAX7 in the DME1/2 enhancers (Himeda *et al*, 2014) could drive *DUX4* expression in satellite cells and muscle progenitors during muscle development, growth and repair (Buckingham & Relaix, 2015). Modelling myogenic specification/commitment in FSHD using embryonic cells and iPSC reveals a peak of DUX4 occurs during a “myogenic progenitor/satellite cell‐like” phase, characterised by increased PAX7 and PAX3 levels (Caron *et al*, 2016; Haynes *et al*, 2017). DUX4 and PAX7 protein were not detected in the same cell however (Haynes *et al*, 2017), but such under‐representation may indicate that DUX4 precludes *PAX7* expression and/or vice versa.

## DUX4 inhibits myogenesis and induces apoptosis

DUX4 can inhibit the functions of myogenic regulatory factors MYOD and MYOGENIN that are required for myogenic differentiation and disrupt the enhancer of *MYF5* (Bosnakovski *et al*, 2008; Bosnakovski *et al*, 2018), as well as indirectly activate HEY1, a myogenic repressor (Young *et al*, 2013). Apoptosis is induced by DUX4 in many cell types and species (Kowaljow *et al*, 2007; DeSimone *et al*, 2020) but exactly how remains unclear. While DUX4 induces p53‐dependent apoptosis (Wallace *et al*, 2010), it drives apoptosis in *TP53*‐null mice too, possibly via upregulation of p21 (Bosnakovski *et al*, 2017a). DUX4 may also force apoptosis by affecting mitochondrial function and sensitising cells to oxidative stress via disruption of the glutathione redox pathway (Bosnakovski *et al*, 2008), induction of reactive oxygen species (ROS) (Dmitriev *et al*, 2016), HIF1α signalling (Banerji *et al*, 2015; Banerji *et al*, 2017; Lek *et al*, 2020) and/or increased c‐MYC and stabilisation of dsRNA (Shadle *et al*, 2017).

Although DUX4 is restricted to apes and Old World monkeys, animal models have been developed to examine the role of DUX4 *in vivo* (DeSimone *et al*, 2020). In particular, mouse models with inducible/controllable DUX4 expression demonstrate hallmarks of FSHD including reduced muscle strength and histopathological features such as muscle atrophy, degeneration, inflammation and fibrosis. Severity of the FSHD‐like pathological phenotype in murine muscle correlates with DUX4 levels (Giesige *et al*, 2018; Jones & Jones, 2018; Bosnakovski *et al*, 2020; Jones *et al*, 2020). Importantly, the differential gene expression profile in *DUX4* over‐expressing mouse muscle has significant overlap with MRI‐guided (inflamed) FSHD muscle biopsies (Bosnakovski *et al*, 2020).

## Deciphering DUX4 function via target gene identification

DUX4 target gene investigation broadly divides into validation or discovery studies. Validation studies typically identify a gene associated with FSHD and determine if it is a DUX4 target gene, as with *PITX1* (Dixit *et al*, 2007), *FRG1* (Ferri *et al*, 2015), *FRG2* (Thijssen *et al*, 2014) and *CRYM* (Vanderplanck *et al*, 2011). Discovery studies often combine expression of *DUX4* with an assay of transcriptomic change, and possibly ChIP‐seq, to identify genes regulated by DUX4 (Young *et al*, 2013; Yao *et al*, 2014).

Discovery studies identify both direct and indirect DUX4 target genes to inform about affected pathways that can then be validated in FSHD. DUX4 discovery studies have been performed in human myoblasts, rhabdomyosarcoma cells, murine myoblasts, murine embryonic stem cells and embryos of *Danio rerio*,*Xenopus laevis* and *Drosophila melanogaster* (DeSimone *et al*, 2020). Comparing transcriptional changes induced by DUX4 in mouse and human show significant correlations in global gene expression changes, particularly in genes associated with cell death and myogenesis (Sharma *et al*, 2013; Knopp *et al*, 2016). However, human cells demonstrate activation of immune‐related, inflammatory gene pathways not found as readily in other species (Geng *et al*, 2012; Sharma *et al*, 2013). Variation in DUX4 target genes between species, and DUX4 being restricted to primates, has led to a focus on DUX4 target gene expression in human.

Three major studies have assayed DUX4 target gene expression in human myoblasts over‐expressing DUX4 for different lengths of time. A set of 212 “early” DUX4 target genes (Banerji *et al*, 2017) was derived after 8 h of *DUX4* expression assayed by RNA‐seq (Choi *et al*, 2016b). A set of 165 “late” DUX4 target genes (Banerji *et al*, 2017) was derived after *DUX4* expression for 24 h using microarray analysis (Geng *et al*, 2012). Finally, another set of 114 “late” DUX4 target genes was identified by fold change via RNA‐seq after 24–48 h of *DUX4* expression and correspondence to ChIP‐seq peaks (Young *et al*, 2013; Yao *et al*, 2014). In addition, 122 endogenous DUX4 target genes were described after 72 h of differentiation in myocytes by performing RNA‐seq and ChIP‐seq (Rickard *et al*, 2015). Gene set enrichment analysis (GSEA) of these 114–212 DUX4 target genes indicates roles in RNA splicing, germ line genes, immune‐related genes and retroelements (Geng *et al*, 2012; Young *et al*, 2013; Rickard *et al*, 2015).

Surprisingly, overlap between transcriptomic and ChIP‐seq data is low. DUX4 target genes have a mean overlap of ~10% (except between Geng *et al*, 2012 and Young *et al*, 2013, where the 27–39% overlap is likely due to similar protocols and using the same ChIP‐seq dataset). The limited 17% overlap between ChIP‐seq peaks at 6 and 24 h of *DUX4* expression may indicate temporal changes in target gene activation. Only eight DUX4 target genes are common to all four studies: *ZSCAN4, TRIM43, RFPL1, RFPL2, RFPL4B, PRAMEF1, PRAMEF2* and *PRAMEF12*.

## DUX4 target genes in FSHD muscle

Very low expression of *DUX4* may translate to uniform induction of DUX4 target genes in FSHD tissue, providing a clear bottom‐up genotype‐to‐phenotype link for understanding pathogenesis. In eight independent FSHD muscle gene expression datasets (totalling 157 FSHD and 98 matched control biopsies), the three “exogenous” DUX4 target gene sets were elevated in only 3/8 datasets (Banerji & Zammit, 2019) (Table 1). Meta‐analysis via random effects modelling, Fisher’s combined *p*‐testing and ROC curve analysis demonstrated that only the “late” (Geng *et al*, 2012) DUX4 target genes had a weak positive association with FSHD (Banerji *et al*, 2017). Evaluation of early and late DUX4 target gene sets on expression data can be easily performed using published software (Banerji & Zammit, 2019).

**Table 1:** Summary of DUX4 and PAX7 target gene signatures in FSHD.

Reference for data access	Sample material	Sample size	Inflammation	Technology	DUX4 transcript detected	DUX4 target genes up in FSHD (Yao, Geng, Choi)	PAX7 target gene score down in FSHD	DUX4 and PAX7 target gene signatures assessed in parallel by
Rahimov *et al* (2012) GSE36398	Muscle biopsies	13 FSHD 12 Ctrl	No	Microarray	No	0	Yes	Banerji *et al* (2017)
Bakay *et al* (2006) GSE3307	Muscle biopsies	14 FSHD1 16 Ctrl	No	Microarray	No	0	Yes	Banerji *et al* (2017)
Tasca *et al* (2012) GSE26852	Muscle biopsies	7 FSHD 7 Ctrl	Subset STIR+	Microarray	No	1/4 signatures (Yao – late)	Yes	Banerji *et al* (2017)
Osborne *et al* (2007) GSE10760	Muscle biopsies	19 FSHD 30 Ctrl			No	0	Yes	Banerji *et al* (2017)
Dixit *et al* (2007) GSE9397	Muscle biopsies	14 FSHD 6 Ctrl	No	Microarray	No	0	Yes	Banerji *et al* (2017)
Yao *et al* (2014) GSE56787	Muscle biopsies	15 FSHD1 8 Ctrl	Transcriptomic evidence	RNA‐seq	No	3/3 signatures (4/4 with Rickard)	Yes	Banerji *et al* (2017)
Wang *et al* (2019) GSE115650	Muscle biopsies	31 FSHD1, 3 FSHD2 9 Ctrl	Subset STIR+	RNA‐seq	No	3/3 signatures (4/4 with Rickard)	Yes	Banerji and Zammit (2019)
Wong *et al* (2020) GSE140261	Muscle biopsies	24 FSHD1, 3 FSHD2 8 Ctrl	Subset STIR+	RNA‐seq	No	3/3 signatures (4/4 with Rickard)	Yes	Banerji (2020)
Banerji *et al* (2017) GSE102812	Immortalised myoblast lines	5 FSHD1 lines 4 Ctrl lines	NA	RNA‐seq	No	2/3 signatures (Yao and Geng – late)	Yes	Banerji *et al* (2017)
Banerji *et al* (2019) GSE123468	Immortalised myotube lines	5 FSHD1 lines 4 Ctrl lines	NA	RNA‐seq	2/15 FSHD samples 0/15 Ctrl samples	3/3 signatures	Yes	Banerji *et al* (2019)
van den Heuvel *et al,* 2019 GSE122873	Single FSHD1/2 myocyte	2 FSHD1, 2 FSHD2 2 Ctrl	NA	scRNA‐seq	27/5133 FSHD 0/1914 Ctrl	3/3 signatures	Yes	Banerji and Zammit (2019)
Jiang et al (2020) GSE143493	Single FSHD2 myonuclei	2 FSHD2 4 Ctrl	NA	snRNA‐seq	3/79 FSHD2‐1, 0/107 FSHD2‐2 0/131 Ctrl	Not assessed	Not assessed	Not assessed
Banerji *et al* (2020c) GSE153523	Primary myoblasts	3 FSHD1 3 Ctrl	NA	RNA‐seq	No	2/3 signatures (Yao and Geng – late)	Yes	Banerji *et al* (2020c)
Banerji *et al* (2020c) GSE153523	Primary myotubes	3 FSHD1 3 Ctrl	NA	RNA‐seq	No	3/3 signatures	Yes	Banerji *et al* (2020c)
Banerji *et al* (2020c) GSE153523	Immortalised lymphoblastoid lines	3 FSHD1 lines 3 Ctrl lines	NA	RNA‐seq	9/9 FSHD samples 2/9 Ctrl samples	3/3 signatures	No	Banerji *et al* (2020c)
Signorelli *et al* (2020) Not yet publically available	Peripheral blood	54 FSHD1 29 Ctrl	NA	RNA‐seq	No	0/3 signatures	No	Signorelli *et al* (2020)

The endogenous DUX4 target gene set (Rickard *et al*, 2015) is significantly elevated in the same 3/8 FSHD muscle biopsy datasets (Table 1). Curiously, endogenous DUX4 target genes were repressed in the FSHD microarray dataset profiling vastus lateralis (Osborne *et al*, 2007), a muscle that typically displays milder pathology and later onset. No association between these endogenous DUX4 target genes and FSHD status was found on meta‐analysis (unpublished observations).

DUX4 target genes were significantly elevated in the 3/8 FSHD muscle biopsy studies profiled by RNA‐seq (Yao *et al*, 2014; Wang *et al*, 2019; Wong *et al*, 2020), while the remaining five studies used microarrays (Bakay *et al*, 2006; Osborne *et al*, 2007; Geng *et al*, 2012; Rahimov *et al*, 2012; Tasca *et al*, 2012) (Table 1), potentially highlighting a technical issue. RNA‐seq and microarray typically have a strong concordance, with correlations in transcript abundances ranging between 0.7 and 0.8, but RNA‐seq outperforms microarray in detection of low‐abundance (less than median) transcripts (Kogenaru *et al*, 2012; Wang *et al*, 2014). It is of note that DUX4 target genes detected by RNA‐seq tend to have less than median expression, making detection less sensitive on the 5/8 FSHD muscle biopsy datasets assayed by microarray (unpublished observations). In contrast, DUX4 target genes identified via microarray (Geng *et al*, 2012) show greater than median expression in FSHD muscle and comprise the only DUX4 target gene set that on meta‐analysis discriminates FSHD from control muscle biopsies profiled in the microarray studies (Banerji *et al*, 2017). Microarrays also detect some differential expression events undetected by RNA‐seq (Kogenaru *et al*, 2012). Microarray analysis however, misses unrepresented genes and < 10% of DUX4 target genes identified by RNA‐seq are lacking from all microarray probe sets on which FSHD muscle was profiled (unpublished observations).

While issues with microarray versus RNA‐seq technologies may contribute to low detection of elevated DUX4 target genes, inflammation may be another factor. The “late” DUX4 target gene set (Yao *et al*, 2014) has significant association with the FSHD microarray study that used MRI (Fig 3) to guide muscle biopsy, with a subset of biopsies being STIR‐positive (Tasca *et al*, 2012). STIR positivity is a transient state thought highly inflammatory and associated with acceleration of disease progression (Monforte *et al*, 2019). Two of the three RNA‐seq studies also used MRI to guide collection of STIR‐positive FSHD muscle biopsies (Wang *et al*, 2019; Wong *et al*, 2020) (Table 1). The third RNA‐seq investigation detected high levels of immune gene expression indicative of inflammation (Table 1), these patients also displayed a relatively high average clinical severity score (Yao *et al*, 2014). Thus, of the 4/8 independent muscle datasets that associate raised DUX4 target gene expression with FSHD, all had either MRI or transcriptomic evidence of inflammation and 3/4 were profiled by RNA‐seq, suggesting that both factors contribute to DUX4 target gene detection (Table 1).

Multivariate analysis on FSHD muscle biopsies reveals association between STIR positivity and DUX4 target gene expression (Banerji & Zammit, 2019). Longitudinal studies allow a more accurate gauge of disease progression (Wong *et al*, 2020), and analysis of 27 FSHD patients over one year found no association between DUX4 target genes and FSHD clinical progression (Banerji, 2020; Wong *et al*, 2020).

## DUX4 association with inflammation

Ectopic expression of *DUX4* in human myocytes drives activation of immune‐related genes (Geng *et al*, 2012), and DUX4 target genes are upregulated in actively inflamed STIR‐positive FSHD muscle biopsies (Banerji & Zammit, 2019). Moreover, a mouse model employing inducible *DUX4* expression demonstrates inflammation and differentially expressed genes that overlap with MRI‐guided STIR‐positive FSHD muscle biopsies (Bosnakovski *et al*, 2020). STIR‐positive FSHD muscle is characterised by lymphocytic infiltrates (Fig 2), particularly endomysial CD8^+^ and perivascular CD4^+^ T lymphocytes (Frisullo *et al*, 2011), with B cells and macrophages in close proximity to CD4^+^ T cells at perivascular sites (Arahata *et al*, 1995): cellular distributions with similarities to myositis.

Polymyositis is characterised by endomysial CD8^+^ T‐cell infiltrates and MHC class I on muscle fibres, with CD8^+^/MHC class I complexes in ~ 20% of biopsies (Dai *et al*, 2010). Such CD8^+^/MHC class I complexing promotes invasion and degeneration of non‐necrotic muscle fibres by cytotoxic T cells (Dai *et al*, 2010). While FSHD biopsies regularly demonstrate endomysial CD8^+^ T‐cell infiltrates, detection of MHC class I on myofibres is inconsistent, with no reports of CD8^+^/MHC I complexes and cytotoxic T‐cell invasion of non‐necrotic myofibres (Munsat *et al*, 1972; Arahata *et al*, 1995). Since DUX4 can suppress MHC class I (Chew *et al*, 2019), it could be that DUX4 protects against cytotoxic T‐cell invasion of myofibres in FSHD.

Like FSHD, dermatomyositis is marked by perivascular CD4^+^ T‐ and B‐cell infiltrates and reduced capillary density (Vattemi *et al*, 2014; Lahoria *et al*, 2016). Dermatomyositis exhibits complement‐mediated endothelial injury and so ischaemic muscle fibre degeneration in a perifascicular distribution (Vattemi *et al*, 2014). Similar pathology is not observed in FSHD.

STIR positivity is associated with DUX4 target gene expression, and D4Z4 epigenetic derepression occurs in non‐muscle cell types, including immune cells (Snider *et al*, 2010; Jones *et al*, 2017). Expression of *DUX4* in thymus (Das & Chadwick, 2016), where T‐cell maturation occurs, may also support a role in immune cell function. DUX4 target gene expression is very low in blood from ~700 individuals until ~ 20 years of age (within typical FSHD onset range) but then increases and persists through life (unpublished observations), suggesting an age‐dependent mechanism, such as telomere shortening (Stadler *et al*, 2013). However, differentially expressed genes or pathways in primary blood‐derived cells do not discriminate FSHD patients from controls (Signorelli *et al*, 2020) (Table 1).

Blood‐derived immortalised B‐lymphoblastoid cell lines from 12 multigenerational families are useful tools to examine the immune system in FSHD (Jacobsen *et al*, 1990). Demethylation at D4Z4 in FSHD B‐lymphoblastoid cell lines is characteristic of FSHD, with good correlation between DNA hypomethylation and D4Z4 repeat length. FSHD B‐lymphoblastoid cell lines also express *DUX4‐fl* and certain DUX4 target genes initially evaluated, namely *ZSCAN4*,*TRIM43* and *MBD3L2* (Jones *et al*, 2017). In a later study, *DUX4* transcripts and both early and late DUX4 target genes were readily detected in FSHD B‐lymphoblastoid cell lines via RNA‐seq (Table 1). In contrast, *DUX4* transcripts were not found in several immortalised FSHD myoblast lines, and late (but not early) DUX4 target genes were upregulated, indicating a transient expression of *DUX4* had occurred. While *DUX4* was detectible in a few (17%) FSHD myotube samples, both early and late DUX4 target genes were upregulated, compatible with a DUX4 pulse in differentiation (Banerji *et al*, 2020c) (Table 1). At the single cell level, 0.5% of FSHD myocytes have a pulse‐like dynamic of *DUX4* expression and associated target genes (Banerji & Zammit, 2019). Thus, *DUX4* and DUX4 target gene expression is robust and continuous in lymphoblastoid cells, but pulsatile in myogenic cells.

To specifically examine inflammation in FSHD muscle, 237 significantly upregulated genes were identified in immortalised FSHD B‐lymphoblastoid cell lines to generate the FSHD Lymphoblast score (Banerji *et al*, 2020c). Meta‐analysis across seven FSHD muscle biopsy studies established that mean expression of these FSHD lymphoblast genes was elevated in FSHD and strongest when MRI was used to guide biopsy selection. Multivariate analysis revealed association of FSHD lymphoblast gene expression with histological detection of inflammation in muscle. Of the 237 genes in the FSHD lymphoblast score, 9 are DUX4 target genes (Banerji *et al*, 2020c). Thus, gene expression changes in blood‐derived FSHD B‐lymphoblastoid cell lines associate with gene expression changes in inflamed FSHD muscle biopsies.

## FSHD/DUX4opathy models

*In vivo* models of FSHD are necessary to both understand pathomechanisms and test potential therapeutics (DeSimone *et al*, 2020). Muscle‐specific *DUX4* expression in mouse can model muscle pathology, including inflammatory changes, e.g. (Bosnakovski *et al*, 2020). However, *DUX4* expression dynamics or levels are not accurate without endogenous human regulatory mechanisms, although this is difficult to achieve (Krom *et al*, 2013; Knopp *et al*, 2016). Furthermore, there are limitations in overlap between DUX4 target genes in mouse and humans (Knopp *et al*, 2016). While clearly informative for understanding aspects of FSHD pathology and testing therapeutics aimed to reduce the effects of DUX4, is “exogenous” DUX4 modelling FSHD, or a novel mouse “DUX4opathy”? Larger and longer‐lived animal models of FSHD are in preparation using inducible DUX4 expression in Göttingen minipigs (Professor Peter L. Jones, personal communication). Xenograft models that graft FSHD muscle (Zhang *et al*, 2014) or cells (Moyle *et al*, 2016; Mueller *et al*, 2019) might be more representative. A caveat is the effect on pathological phenotypes of using immunocompromised mouse hosts to avoid rejection of the human tissue/cells, although immune reconstituted mice may mitigate this.

## Working top down: phenotype to genotype

Applying the bottom‐up approach of relating genotype to phenotype by linking DUX4 to FSHD tissue has yielded an informative, yet incomplete, understanding of pathology. An alternative is a “top‐down” approach connecting phenotype to genotype by identifying molecular perturbations in FSHD cells/tissue that correlate with pathology and then investigating the link to the D4Z4 region and/or DUX4. Two of the most elucidated FSHD phenotypes are oxidative stress sensitivity and defective myogenic differentiation.

## Oxidative stress sensitivity and the glutathione redox pathway

FSHD muscle exhibits changes in generation and control of ROS, and oxidative stress sensitivity is a key feature of FSHD myoblasts, which more readily undergo apoptosis on exposure to oxidative stressors *ex vivo* (Winokur *et al*, 2003b; Laoudj‐Chenivesse *et al*, 2005; Celegato *et al*, 2006; Bosnakovski *et al*, 2008; Tsumagari *et al*, 2011; Turki *et al*, 2012; Dmitriev *et al*, 2016). FSHD myoblasts display elevated intracellular ROS and increased DNA damage (Dmitriev *et al*, 2016), while anti‐oxidants can reduce apoptosis (Bosnakovski *et al*, 2008; Denny & Heather, 2017). Moreover, anti‐oxidants were the most frequent class of compounds found in a screen for inhibitors of DUX4‐mediated toxicity (Bosnakovski *et al*, 2014; Choi *et al*, 2016a). On the systemic scale, a clinical trial dosing FSHD patients with anti‐oxidants (vitamin C, E, zinc gluconate and selenomethionine) for 17 weeks showed improvement in maximal voluntary contraction of quadriceps, but no significant effects were reported for the primary outcome measure of the 2‐minute walk test (Passerieux *et al*, 2015). A more recent trial used nutraceutical supplementation of multiple anti‐oxidant and anti‐inflammatory compounds (FLAVOMEGA). However, due to the low numbers enrolled, FSHD and LGMD patients were analysed as a single group, making it unknown if the reported efficacy in primary and secondary endpoints pertained to FSHD (Sitzia *et al*, 2019).

There are a number of proposed mechanisms for oxidative stress sensitivity in FSHD. FSHD myoblasts, muscle and serum all have perturbation of the glutathione redox pathway, resulting in accumulation of oxidised glutathione disulphide and decreased levels of reduced glutathione (Winokur *et al*, 2003a; Vanderplanck *et al*, 2011; Turki *et al*, 2012; Denny & Heather, 2017). Glutathione reduces H_2_O_2_ to water and glutathione disulphide in a reaction catalysed by glutathione peroxidase. Thus, an increase in the glutathione disulphide:glutathione ratio drives accumulation of H_2_O_2_ and related ROS promoting cell and DNA damage (Esteve *et al*, 1999). Impaired muscle oxygenation in FSHD may also contribute to ROS sensitivity (Olivier *et al*, 2016; Wilson *et al*, 2018). Other molecular mechanisms underlying oxidative stress sensitivity include p21 upregulation (Winokur *et al*, 2003a), increased HIF1α (Tsumagari *et al*, 2011; Banerji *et al*, 2015; Banerji *et al*, 2017; Lek *et al*, 2020), mitochondrial dysfunction (Turki *et al*, 2012; Banerji *et al*, 2019), RAGE‐NF‐κB signalling (Macaione *et al*, 2007) and membrane repair deficits (Bittel *et al*, 2020). Compensatory mechanisms for oxidative stress are also enhanced in FSHD, an example being increased catalase to remove H_2_O_2_ (Turki *et al*, 2012; Yao *et al*, 2014).

As discussed, a genotype‐to‐phenotype link to DUX4 is found as DUX4 affects mitochondrial function, induces ROS and sensitises cells to oxidative stress via disruption of the glutathione redox pathway and HIF1α signalling (Bosnakovski *et al*, 2008; Banerji *et al*, 2015; Dmitriev *et al*, 2016; Banerji *et al*, 2017; Lek *et al*, 2020). Interestingly, oxidative stress, in turn, can induce expression of DUX4 in a patient‐derived iPSC model (Sasaki‐Honda *et al*, 2018).

## Defective myogenic differentiation

A second major FSHD phenotype is defective myogenesis (Winokur *et al*, 2003b; Barro *et al*, 2010; Broucqsault *et al*, 2013). Some primary or immortalised clones of FSHD myoblasts demonstrate distinct morphological phenotypes after differentiation *ex vivo*: described as “disorganised’ or “atrophic/hypotrophic” myotubes (Barro *et al*, 2010; Banerji *et al*, 2019). Disorganised FSHD myotubes possess rounded boundaries and abnormal clustering of nuclei and occurred in 43% (6/14) of FSHD patients (Barro *et al*, 2010; Tassin *et al*, 2012), although lack of quantifiable metrics limits categorisation.

The atrophic FSHD myotube phenotype was defined as an increased proportion of myotubes with a diameter less than 20 μm and reduced proportion of myotubes greater than 100 μm in diameter (Barro *et al*, 2010) occurring in 42–57% of FSHD patients (Barro *et al*, 2010; Banerji *et al*, 2019). Using time‐lapse imaging combined with a high‐throughput, image‐quantification algorithm, the smaller FSHD myotubes were found to have never achieved the size of controls, and so were actually hypotrophic, rather than atrophic (Banerji *et al*, 2019).

RNA‐seq at eight morphologically defined time points during FSHD hypotrophic myogenesis identified suppression of mitochondrial biogenesis pathways as prominent, notably peroxisome proliferator‐activated receptor gamma coactivator 1‐alpha (PGC1α) and oestrogen‐related receptor A (ERRα). Knockdown of PGC1α in control myoblasts causes myotube hypotrophy, while ERRα phytoestrogen agonists restore a “healthy” myotube phenotype to FSHD myoblasts (Banerji *et al*, 2019).

The oestrogen receptor ERβ is also suppressed in FSHD hypotrophic myotubes, and exogenous oestrogens restore myotube phenotype (Teveroni *et al*, 2017). This is a consideration given the use of the weakly oestrogenic phenol red (Welshons *et al*, 1988) in culture medium, that may mask more subtle FSHD hypotrophic phenotypes. Demonstration of a role for oestrogens in improving myogenic differentiation in FSHD is consistent with clinical evidence that females tend to have later onset than males and are often less affected (Zatz *et al*, 1998). However, lifetime endogenous oestrogen exposure calculations revealed that disease severity in FSHD was unaltered across periods of hormonal change including menarche, pregnancy or menopause (Mul *et al*, 2018).

A genotype‐to‐phenotype link has been described as ectopic DUX4 drives an atrophic phenotype, while a truncated D4Z4 region or ectopic expression of DUX4c results in disorganised myotubes. It is of note that culture conditions can also affect expression of *DUX4*. For example, *DUX4* is suppressed by dexamethasone (Pandey *et al*, 2015), which may in turn inhibit the atrophic/hypotrophic phenotype. DUX4 affecting MYOD and MYOGENIN function is likely part of the pathomechanism (Bosnakovski *et al*, 2008; Bosnakovski *et al*, 2018). Increased microtubular network and myofibrillar modelling proteins and/or activation of known regulators of muscle atrophy Atrogin1 and MuRF1 have also been implicated in FSHD myogenesis (Yip & Picketts, 2003; Vanderplanck *et al*, 2011; Knopp *et al*, 2016).

## FSHD muscle gene expression

Early transcriptomic studies of FSHD muscle focused on differentially expressed genes, identifying perturbations in processes including myogenesis (Winokur *et al*, 2003b), vascular remodelling (Osborne *et al*, 2007) and inflammation (Tasca *et al*, 2012). Another study reported no transcriptomic changes (Rahimov *et al*, 2012) and no overlap in differentially expressed genes across multiple studies were found (Banerji *et al*, 2015). Indeed, a proposed FSHD biomarker based on 15 differentially expressed genes (Rahimov *et al*, 2012) did not validate on meta‐analysis across five independent FSHD muscle biopsy studies (Banerji *et al*, 2017).

Differential expression appears insufficient to detect perturbations in gene expression and signalling pathways in FSHD muscle. Therefore, the InSpiRe algorithm was developed to investigate information theoretic measures of biological signal transduction on protein interaction networks, weighted with gene expression data from FSHD muscle and cells. The resulting gene set was further refined using expression data from other muscle diseases, ultimately identifying 164 genes specifically rewired in their interactions in FSHD (Banerji *et al*, 2015). Affected pathways included some known to be changed in FSHD, such as TNFα signalling (Turki *et al*, 2012) and Wnt/β‐catenin signalling (Block *et al*, 2013) but also novel pathways including HIF1α: findings consistent across independent datasets (Banerji *et al*, 2015). Some of these top‐down discoveries were also linked to the FSHD genotype via DUX4 over‐expression in murine primary satellite cells (Banerji *et al*, 2015).

A recent study combined a multiobjective genetic algorithm with a multiplex network describing many layers of known biological relation between genes. The MOGAMUN algorithm identified highly connected sub‐networks of differentially expressed genes in the FSHD muscle transcriptomic data of Yao *et al*, 2014, including perturbation of a gene module involved in mitochondrial function centred around 2‐oxoglutarate dehydrogenase‐like, mitochondrial (OGDHL). Although no active modules contained DUX4 target genes (possibly due to poor curation of relationships between these genes), 23 active modules involving PAX7 target genes were found (preprint: Novoa‐del‐Toro *et al*, 2020).

## The PAX7 target gene score is a powerful biomarker for FSHD: from the top down

Similarity in sequence and function of the homeodomains of DUX4 and PAX7 (Bosnakovski *et al*, 2008; Bosnakovski *et al*, 2017b), that PAX3/7 can ameliorate the DUX4‐mediated deleterious phenotype in mouse myoblasts (Bosnakovski *et al*, 2008), and PAX7 being a master regulator of post‐natal myogenesis (Seale *et al*, 2000), prompted us to further examine the role of PAX7 in FSHD. DUX4 predominately activates its target genes, while PAX7 both activates and suppresses target genes (Buckingham & Relaix, 2015). We therefore constructed a single sample PAX7 target gene score comprising 311 upregulated and 290 downregulated PAX7 target genes from analysis of murine satellite cell‐derived myoblasts over‐expressing PAX7 or a dominant‐negative PAX7‐ERD fusion (Banerji *et al*, 2017). The PAX7 target gene score can be computed using published software (Banerji & Zammit, 2019). These PAX7 target genes are likely both direct and indirect targets, but PAX7 ChIP (Lilja *et al*, 2017) could be employed to determine the proportion of each.

The PAX7 target gene score, although not PAX7 itself, is significantly repressed in each of eight transcriptomic FSHD muscle biopsy studies, being unaffected by microarray/RNA‐seq technology or STIR positivity indicating inflammation (Banerji & Zammit, 2019) (Table 1). Repression of the PAX7 target gene score further correlates with histopathological markers of FSHD severity (pathology score, inflammation and active disease) independently of DUX4 target gene expression. Moreover, FSHD muscle biopsies with both high DUX4 target gene expression and robust PAX7 target gene score repression display more than twice the amount of histologically active disease compared to those with only high DUX4 target genes and minimal/no PAX7 target gene score repression, showing that DUX4 target genes are not the sole driver of disease. Importantly, a lower PAX7 target gene score associates with FSHD clinical outcome correlating with fatty replacement of muscle (T1 positivity on MRI – Fig 3) independently of DUX4 target gene activation. The PAX7 biomarker is not associated with the transient inflammatory state of STIR positivity (Fig 3) so not confounded by fluctuating inflammation, unlike DUX4 target gene expression (Banerji *et al*, 2017; Banerji & Zammit, 2019; Banerji, 2020). Thus, DUX4 and PAX7 target gene scores independently correlate with markers of active disease, but DUX4 is associated with transient inflammation, so is a less stable marker of disease progression. PAX7 target gene score repression is not a general feature of dystrophic muscle, as shown by examining muscle biopsies from Duchenne muscular dystrophy (Banerji *et al*, 2017).

Repression of the PAX7 target gene score is a reliable biomarker of FSHD myocytes *ex vivo*. This contrasts to DUX4 target genes, which are only detectable in ~ 23% of FSHD myocytes (Banerji & Zammit, 2019). Importantly, unlike DUX4 target gene expression, PAX7 target gene score repression does not associate with FSHD B‐lymphoblastoid cell lines, implying a muscle specific FSHD biomarker (Banerji *et al*, 2020c).

In a longitudinal study of FSHD muscle via MRI, transcriptomics and histology (Wong *et al*, 2020), PAX7 target gene score repression was significantly associated with FSHD patient progression over one year (Banerji, 2020). This was in contrast to DUX4 target gene expression, MRI and histopathology that were not associated with disease progression (Banerji, 2020; Wong *et al*, 2020). Alas, the ongoing 18‐month longitudinal “ReSolve” study (NCT03458832) of 220 FSHD patients to validate two novel clinical outcome assessments as clinical trial tools does not include muscle biopsy (LoRusso *et al*, 2019).

## PAX7 target gene score repression and muscle regeneration

*PAX7* is ubiquitously expressed in quiescent and activated human and murine satellite cells (Reimann *et al*, 2004; Zammit *et al*, 2004; Marg *et al*, 2014). Satellite cells proliferate to provide myoblasts that differentiate and fuse to replace myonuclei: redeploying many regulatory pathways used during developmental myogenesis (Zammit, 2017). The central role played by PAX7 in regenerative myogenesis is shown by genetic deletion, which severely affects specification of satellite cells and is essential for muscle regeneration in mouse (Seale *et al*, 2000). In humans, loss‐of‐function *PAX7* mutations lead to the congenital muscular dystrophy MYOSCO (OMIM: 618578) with similarities to some cases of early onset FSHD including hypotonia, muscular atrophy, decreased respiratory function and spinal deformities (Mah & Chen, 2018; Feichtinger *et al*, 2019; Goselink *et al*, 2019a; Marg *et al*, 2019). Unlike MYOSCO though, the single report available indicates that the median satellite cell number is unchanged in 10 adult FSHD patients (18–75 years of age) (Statland *et al*, 2015).

A regenerative response is evident in many muscular dystrophies, e.g. Duchenne muscular dystrophy (Janghra *et al*, 2016). Progressive muscle weakness and wasting however, shows that any repair response eventually fails to keep pace with dystrophic changes. This is, in part, because satellite cells operate in an increasingly hostile microenvironment with chronic inflammation and fibrosis. However, the pathogenic mutation that elicits myofibre damage may also directly affect satellite cell function to further compromise any regenerative response (Morgan & Zammit, 2010; Morgan & Partridge, 2020).

Linking to genotype in FSHD, DUX4 is functional in primary satellite cell‐derived myoblasts from FSHD patients (Rickard *et al*, 2015) and detectable in myogenic cells during muscle regeneration in the D4Z4‐2.5 mouse, transgenic for a 2.5 D4Z4 unit region from an FSHD patient (Krom *et al*, 2013; Knopp *et al*, 2016). DUX4 may also alter satellite cell function via epigenomic changes made from expression earlier in embryogenesis/foetal development/growth (Ferreboeuf *et al*, 2014; Haynes *et al*, 2017).

## Extent of muscle regeneration in FSHD

Transcriptomic and proteomic studies indicate downregulation of MYOD and MYOD‐dependent gene networks in FSHD muscle that would affect regenerative myogenesis (Winokur *et al*, 2003b; Celegato *et al*, 2006). In addition, a subset of FSHD muscle biopsies display a “muscle‐low” transcriptome, suppressing genes typically involved in myogenesis (Wong *et al*, 2020). Hypotrophic or disorganised FSHD myotubes further show that myogenesis is perturbed (Barro *et al*, 2010; Banerji *et al*, 2019).

Regenerating muscle fibres in FSHD muscle were originally identified using histology (Padberg, 1982; Lin & Nonaka, 1991; Arahata *et al*, 1995) and then by developmental MyHC immunolabelling (Rogers *et al*, 2004; Celegato *et al*, 2006). Regenerating fibres were generally rare however, for example, found in only 3/11 FSHD deltoid biopsies (Celegato *et al*, 2006). Such observations contributed to the perception that muscle regeneration was not a feature of FSHD.

To quantify muscle regeneration in FSHD, we recently reported a large‐scale systematic investigation (Banerji *et al*, 2020b). At the transcriptomic level, a signature describing muscle regeneration using mean expression of 200 human genes (HALLMARK_MYOGENESIS) (Subramanian *et al*, 2005) was significantly elevated in FSHD muscle, as well as in Duchenne muscular dystrophy and Myotonic dystrophy type 2. Refinement of this transcriptomic signature to include more recently described genes central to human myogenic differentiation such as *MYOMAKER* and *MYOMIXER* (Zhang *et al*, 2020), should add to the power.

Developmental MyHC isoform‐containing regenerating myofibres (Fig 2) were found in most (77%) FSHD quadriceps from adult patients at a mean of 0.5% regenerating myofibres per biopsy and in the vast majority (91%) of tibialis anterior muscles, averaging 1.7% myofibres per biopsy. Regenerating myofibres associate with pathological severity, including the pathogenic hallmarks of fibre size variation, central nucleation and fibrosis (Banerji *et al*, 2020b). For comparison, the equally slowly progressing Myotonic dystrophy type 2 had a similar proportion of regenerating muscle fibres per biopsy (1.2%).

Serum creatine kinase levels reflect myofibre damage and are usually normal or only slightly raised in FSHD (Padberg, 1982). In contrast, Duchenne muscular dystrophy generally exhibits high serum creatine kinase levels with severe pathology in many muscles (Grounds *et al*, 2020). This is accompanied by 24–47% regenerating fibres in young boys (Decary *et al*, 2000; Janghra *et al*, 2016; Scaglioni *et al*, 2020), compared to ~ 1% in healthy growing boys (Scaglioni *et al*, 2020). FSHD is similar to most muscular dystrophies then, in mounting a regenerative response to chronic dystrophy, but regeneration may be compromised, e.g. (Corasolla Carregari *et al*, 2021). Regenerative therapies to enhance muscle repair in FSHD could mitigate muscle damage and atrophy.

## Other consequences of PAX7 target gene score repression

Functionally, PAX7 promotes cell survival and proliferation, while preventing precocious myogenic differentiation (Relaix *et al*, 2006; Collins *et al*, 2009). GSEA shows that PAX7 target gene score repression associates with activation of HIF1α target genes (Banerji *et al*, 2017): a mechanism identified in both top‐down studies of FSHD muscle (Banerji *et al*, 2015) and differentiating myoblasts (Tsumagari *et al*, 2011), and bottom‐up approaches via DUX4 toxicity (Lek *et al*, 2020). HIF1α activation likely contributes to oxidative stress sensitivity and may contribute to aberrant vasculature via induction of angiogenic factors such as VEGF.

PAX7 target gene score repression also associates with upregulation of EZH2 target genes (Banerji *et al*, 2015). EZH2 facilitates epigenetic silencing and is at the D4Z4 region of healthy individuals, but not FSHD patients (Cabianca *et al*, 2012). PAX7 also drives long‐term epigenetic changes associated with derepression of gene expression. This is by affecting both DNA demethylation (Carrio *et al*, 2016) and chromatin. Localised chromatin is remodelled by PAX7 via induction of chromatin accessibility and histone marks associated with active enhancers (Lilja *et al*, 2017). PAX7 can operate through the Wdr5‐Ash2L‐MLL2 histone methyltransferase complex that directs H3K4 methylation (McKinnell *et al*, 2008).

## Linking genotype to phenotype: interaction of DUX4 and PAX7

*DUX4* enhancers DME1/2 contain binding sites for PAX3 and PAX7 (Himeda *et al*, 2014). Modelling early stages of myogenesis using FSHD embryonic stem cells and iPSCs reveal an increase in *DUX4* during a myogenic progenitor/satellite cell phase characterised by increased PAX7 and PAX3 levels. Significantly, PAX7 and DUX4 were not detected in the same cell by immunolabelling at the few time points assayed, indicative of possible mutual preclusion (Caron *et al*, 2016; Haynes *et al*, 2017). *DUX4* is expressed in human satellite cell‐derived myoblasts isolated from adult FSHD muscle, with levels then increasing during differentiation (Tassin *et al*, 2013; Balog *et al*, 2015; Rickard *et al*, 2015).

The significant amino acid sequence similarity of the homeodomains of DUX4 to those of PAX3 and PAX7 prompted the hypothesis that competitive inhibition of PAX7 target gene activation by DUX4 may contribute to FSHD (Bosnakovski *et al*, 2008). Indeed, the homeodomain of PAX7 or PAX3 can substitute those of DUX4 without drastically affecting DUX4‐driven myoblast apoptosis and inhibited myogenesis (Bosnakovski *et al*, 2017b). Over‐expression of Pax3/7 in murine myoblasts also rescues DUX4‐mediated cytotoxicity (Bosnakovski *et al*, 2008). Moreover, inhibition is reciprocal, with co‐expression of exogenous PAX7 and DUX4 in human cells leading to mutual suppression of their respective transcriptional target genes (Banerji *et al*, 2017). DUX4‐mediated inhibition of PAX7 target gene activation, however, is greater than expected from a competitive DNA‐binding model, suggesting that DUX4 may also potentially operate by dimerisation, sequestering co‐factors or direct repression of PAX7. It is notable that DUX4 induction of apoptosis involves activation of caspases (Banerji *et al*, 2015), which also directly cleave and inactivate PAX7 in murine satellite cells (Olguin, 2011; Dick *et al*, 2015).

PAX7 has 86% sequence similarity to PAX3. Pax3 is expressed in a subset of satellite cells in particular mouse muscles (Calhabeu *et al*, 2013), with a role in protecting against environmental toxins (Der Vartanian *et al*, 2019). The extent of PAX3 expression in human satellite cells is unknown, as is its regional distribution, but PAX3 could influence the range of muscles affected in FSHD and their regenerative potential, especially given its role in dealing with toxins.

## Summary: bottoms up

The bottom‐up approach has recently focused on understanding the role of DUX4, which perturbs immune gene expression (Yao *et al*, 2014), inhibits myogenesis (Bosnakovski *et al*, 2008) and drives apoptosis (Kowaljow *et al*, 2007). However, *DUX4* is very difficult to detect in FSHD muscle (Tassin *et al*, 2013). Surprisingly, DUX4 target genes do not show great concordance in different studies, and no DUX4 target gene signature is a consistent FSHD biomarker (Banerji *et al*, 2017).

DUX4 target gene expression in FSHD muscle mainly associates with two factors. First, experimental technology: DUX4 target genes are elevated in muscle biopsies analysed by RNA‐seq (3/3), but less so by microarray (1/5) (Banerji & Zammit, 2019) (Table 1). Second, inflammation: DUX4 target genes are elevated in all STIR‐positive muscle biopsy datasets (2 RNA‐seq/1 microarray) and a dataset with transcriptomic evidence of inflammation (Banerji & Zammit, 2019) (Table 1). Since the 3 RNA‐seq datasets from FSHD muscle biopsies showed signs of inflammation (Table 1), the DUX4 associated factors of technology and inflammation are confounded, making it unknowable whether associations are independent.

**Figure 4:**
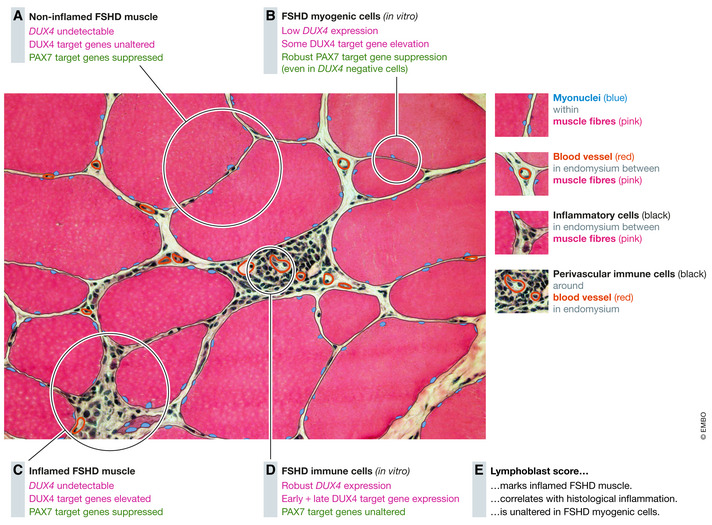
Summary of DUX4 and PAX7 target gene expression in FSHD Graphic illustration of how DUX4 and PAX7 target genes associate with FSHD muscle. Schematic render of Fig 2B that demonstrates characteristic FSHD pathology, including endomysial and perivascular inflammation. (A and C) DUX4 target genes are only reliably detected in actively inflamed FSHD muscle biopsies using DUX4 target gene signatures. (B) DUX4 and DUX4 target genes are expressed in a small proportion of primary and immortalised myoblast cell lines derived from FSHD patients. (D) In contrast, DUX4 and DUX4 target genes are robustly expressed in FSHD patient blood‐derived immortalised B‐lymphoblastoid clones. (A and C) The PAX7 target gene signature shows that PAX7 target genes are suppressed in all FSHD muscle biopsies regardless of inflammatory status, suggesting a more muscle specific effect. (B) Suppression of PAX7 target genes marks the majority of primary and immortalised FSHD myoblast cell lines. (D) PAX7 target genes are not suppressed in the FSHD B‐lymphoblastoid clones. (E) The lymphoblast score, composed of 237 genes upregulated in immortalised FSHD B‐lymphoblastoid cell lines, distinguishes FSHD from control muscle biopsies and is strongest when MRI is used to guide biopsy selection.

However, DUX4 target gene expression correlates with STIR positivity and histological inflammation (Fig 4). High *DUX4* and DUX4 target gene expression are also found in blood‐derived immortalised FSHD B‐lymphoblastoid immune lines, while FSHD myogenic lines display very low and transient levels (Banerji *et al*, 2020c). Persistence of macrophages in an inducible *DUX4* mouse model indicates perturbed inflammation (Bosnakovski *et al*, 2020). *DUX4* transcripts are also detectable in immune cells in secondary lymphoid organs (inguinal lymph nodes and spleen) in the D4Z4‐2.5/Dnmt3b^MommeD14^ and D4Z4‐2.5/Smchd1^MommeD1^ mouse models of FSHD (Bouwman *et al*, 2020). Specific immune cell populations are changed in the spleen of D4Z4‐2.5/Smchd1^MommeD1^ mice including an increase in B cells and reduction of T and myeloid cells (Bouwman *et al*, 2020). To better understand how DUX4 affects function of the immune system, further examination of DUX4 and its target genes in muscle infiltrating immune cells from FSHD patients is required. Direct comparisons of STIR‐positive/negative areas/muscles in the same FSHD patients would be informative, as would proteomics on microdialysis from muscle interstitium (Corasolla Carregari *et al*, 2021). Inflammation accelerates fatty replacement, which nevertheless occurs at a slower rate in its absence, and macroscopic inflammation can resolve without fatty replacement. Thus, therapies aimed at DUX4 and DUX4 target genes may prevent acceleration of FSHD progression via inflammation but may not fully address muscle fatty replacement. Importantly, although well correlated to MRI measures of inflammation, DUX4 target gene expression is unrelated to clinical progression (Banerji, 2020; Wong *et al*, 2020).

DUX4 in non‐FSHD pathology suggests that it may be anti‐apoptotic and a driver of survival, proliferation and immune evasion in multiple cancers. This contrasts with pro‐apoptotic and anti‐myogenic effects causing inflammatory and degenerative pathology in FSHD muscle. DUX4 suppresses MHC class I in cancer (Chew *et al*, 2019), and so may also be protective in FSHD by preventing infiltration of non‐necrotic fibres by endomysial CD8^+^ T‐cell infiltrates, as occurs in polymyositis. A concern, therefore, is that anti‐DUX4 therapy (Le Gall *et al*, 2020; Schatzl *et al*, 2021) may facilitate immune cell‐mediated myofibre damage, as well as have effects on normal bone and skin homeostasis (de la Kethulle de Ryhove *et al*, 2015; Gannon *et al*, 2016).

*DUX4* and a subset of its target genes are detected in foetal human FSHD muscle (Broucqsault *et al*, 2013; Ferreboeuf *et al*, 2014) and *DUX4* is expressed in iPSCs FSHD models of early myogenic specification and myogenesis (Haynes *et al*, 2017). Expression early in development means that DUX4‐mediated effects could then be slowly accumulative and/or DUX4 causes epigenetic changes that manifest as dystrophy/defective regeneration later in life, in the absence of robust DUX4 levels. The stochastic and pulsatile expression of *DUX4* in adult‐derived FSHD myoblasts and differentiated myogenic cells *in vitro* (Table 1) does indicate a direct effect of DUX4 in muscle fibres and on regenerative myogenesis (Banerji *et al*, 2020b). Persistence of FAPs, M1 and M2 macrophages after DUX4 induction in mouse (Bosnakovski *et al*, 2020) would also impact on the coordination of muscle regeneration. Expression dynamics of *DUX4* during development and growth requires further examination, as does how DUX4 contributes to pathological damage.

## Summary: view from the top

Top‐down studies show that FSHD myogenic cells exhibit oxidative stress sensitivity (Winokur *et al*, 2003a) and impaired myogenesis (Winokur *et al*, 2003b; Bosnakovski *et al*, 2008). Oxidative stress sensitivity is also observed in *DUX4*‐expressing myoblasts (Bosnakovski *et al*, 2008), providing a genotype‐to‐phenotype link, and anti‐oxidant therapies have been clinically trialled in FSHD (Passerieux *et al*, 2015). However, the cause and mechanistic nature of oxidative stress is lacking, as is the source of elevated ROS levels. Impaired myogenesis in FSHD can be identified by a hypotrophic myotube phenotype, linked to suppression of PGC1α/ERRα (Banerji *et al*, 2019) and ERβ (Teveroni *et al*, 2017). Phytoestrogens target both pathways and can rescue hypotrophy (Banerji *et al*, 2019). Identification of impaired mitochondrial biogenesis as a driver of hypotrophy also links oxidative stress sensitivity and defective myogenesis, suggesting a common therapeutic target in mitochondria. Yet, connection between the mitochondrial defect and *DUX4* expression is lacking. The transcriptome/proteome of FSHD and *DUX4*‐expressing cells under oxidative stress with/without anti‐oxidants is required.

Limitations of poor concordance in differentially expressed genes have been overcome by application of sophisticated, bioinformatic, network‐based tools. These have identified consistent signals in pathway disruption in FSHD muscle biopsies, including TNFα signalling, HIF1α over‐activation, Wnt/β‐catenin signalling and mitochondrial function via OGDHL (Banerji *et al*, 2015; preprint: Novoa‐del‐Toro *et al*, 2020).

Top‐down examination also shows that PAX7 target gene score repression is a significant biomarker of FSHD status and associated with FSHD muscle cells (Banerji & Zammit, 2019) but not FSHD immune cells (Banerji *et al*, 2020c) (Fig 4). Importantly, PAX7 target gene score repression progresses in FSHD patient muscle over a year, a unique feature not achieved by *DUX4*, DUX4 target gene expression or non‐transcriptomic markers (Banerji, 2020). PAX7 target gene score repression also associates with histological metrics of FSHD disease activity and fatty replacement seen on MRI in a manner independent of DUX4 target gene activation but is not associated with inflammation (Fig 4).

Inhibition of PAX7 function by transient DUX4 expression in development and/or regeneration may result in global perturbation of PAX7 transcriptional target genes. This could be indirect, via the actions of DUX4 transcriptional target genes, or by direct interference of DUX4 with PAX7. This would not only affect muscle progenitor cells, but could also have effects in muscle fibres as PAX7 can reconfigure the epigenome through DNA demethylation (Carrio *et al*, 2016) and the Wdr5‐Ash2L‐MLL2 histone methyltransferase complex (McKinnell *et al*, 2008). Interestingly, *DUX4* is not co‐expressed with PAX7 in iPSCs during myogenesis, interpreted as meaning that they are expressed at different stages (Haynes *et al*, 2017). However, it could also mean that DUX4 and PAX7 are precluded from co‐expression or that co‐expression leads to rapid cell death. Homeodomain‐containing proteins can also cross cell membranes, so DUX4 and PAX7 may not even require expression in the affected cell (Lee *et al*, 2019).

Perturbation of PAX7 target genes would affect satellite cell‐mediated muscle growth, homeostasis and repair, making myonuclear turnover/microtrauma repair less efficient in FSHD. The number of muscle fibres is established during early human foetal development, with growth rapid in the perinatal period, before slowing during childhood. Growth by both increase in myonuclei number and myofibre size accelerates again during adolescence, continuing up to around age 15 in girls and 18 years for boys (Partridge, 2013). Since most FSHD patients are asymptomatic in childhood/early adolescence, this raises the question of whether developing/growing muscle is better able to respond to DUX4 or factors such as oxidative stress, rendering any effects initially subclinical. When satellite cells become mitotically quiescent as muscle matures in late adolescence however, such damage may accumulate more rapidly or epigenetic changes have more effect, leading to symptoms. It is of note that in the *mdx* mouse model of Duchenne muscular dystrophy, myonuclear accretion from satellite cells falls drastically around 3 weeks of age (White *et al*, 2010; Bachman *et al*, 2018), coinciding with a first wave of muscle degeneration (Bulfield *et al*, 1984).

Although the PAX7 target gene score is a clear biomarker of FSHD status and progression (Banerji, 2020), association between PAX7 target gene score repression and a targetable pathological process is still being elucidated. However, important roles seem apparent for PAX7‐regulated HIF1α over‐activation and muscle regeneration.

## Conclusions: meeting in the middle

Bottom‐up and top‐down approaches reveal two major mechanisms. DUX4 target gene expression seems associated with muscle that exhibits transient, unpredictable inflammation, which often accelerates muscle degeneration (Fig 4). Conversely, PAX7 target gene score repression associates with persistent degeneration of FSHD skeletal muscle, which occurs predictably and gradually in FSHD, even in the absence of overt inflammation (Fig 4). Both mechanisms are independently associated with FSHD active pathology. A full understanding of FSHD pathology and appropriate therapy will be incomplete if only one is considered.

## Author contributions

PSZ and CRSB contributed equally.

## Conflict of interest

The authors declare that they have no conflict of interest.

**Pending issues**

- Accurately model endogenous *DUX4* expression: quantification of the biological half‐life of DUX4, combined with determination of the diffusion radius of DUX4 in a muscle fibre syncytium (and beyond), is needed to estimate how many myonuclei are expressing DUX4 at any one time.
- Directly compare transcriptome/proteome data between STIR^+^ and STIR^‐^ FSHD muscle biopsies and immune cells from the same patient to account for patient heterogeneity.
- Understand muscle regeneration in FSHD muscle to determine whether it is scarce due to low stimulus and/or being directly compromised by DUX4/PAX7 and FSHD pathology.
- Investigate metabolism and oxidative stress in FSHD as a major feature of pathology.
- Examine redox control over DUX4 and PAX7 gene expression.
- Characterise interactions between DUX4 and PAX7.
- Identify the pathological consequence of perturbation of PAX7 target genes and which are the crucial genes/pathways perturbed.
- Comprehend what underlies inter‐patient heterogeneity, male/female differences and left/right asymmetry in FSHD via examining parameters including modifying genes, hormones, telomere shortening, miRNAs and lncRNAs.
- Accurately delimit which aspects of FSHD pathology and therapeutic testing that can be accurately modelled in DUX4opathy and non‐primate animal models.

**For more information**

- Muscular Dystrophy UK – https://www.musculardystrophyuk.org/about‐muscle‐wasting‐conditions/facioscapulohumeral‐muscular‐dystrophy‐fsh/
- FSHD Society – https://www.fshdsociety.org
- Software to evaluate the 3 DUX4 target gene signatures (Geng *et al*, Yao *et al* and Choi *et al*) and the PAX7 target gene signature is available at: https://academic.oup.com/hmg/article/28/13/2224/5376488#supplementary‐data
- MyFSHD – https://myfshd.org
- UK FSHD Patient Registry – https://www.fshd‐registry.org.uk
- National Registry for Myotonic Dystrophy (DM) & Facioscapulohumeral Dystrophy (FSHD) – https://www.urmc.rochester.edu/neurology/national‐registry.aspx
- Italian National Registry for Facioscapulohumeral Muscular Dystrophy – https://treat‐nmd.org/patient‐registry/fshd‐registry‐italy/
- FSHD registry France – https://treat‐nmd.org/patient‐registry/fshd‐registry‐france/
- FSHD cell source – https://www.umassmed.edu/wellstone/
- FSHD cell source – https://www.institut‐myologie.org/en/
